# Annual global dengue dynamics are related to multi-source factors revealed by a machine learning prediction analysis

**DOI:** 10.1371/journal.pntd.0013232

**Published:** 2025-06-25

**Authors:** Haoyu Long, Yilin Chen, Jingru Feng, Jian Chen, Xue Zhang, Wenjie Han, Min Kang, Xiangjun Du

**Affiliations:** 1 School of Public Health (Shenzhen), Sun Yat-sen University, Guangzhou, P.R. China; 2 School of Public Health (Shenzhen), Shenzhen Campus of Sun Yat-sen University, Shenzhen, P.R. China; 3 Guangdong Provincial Center for Disease Control and Prevention, Guangzhou, P.R. China; 4 Shenzhen Key Laboratory of Pathogenic Microbes & Biosafety, Shenzhen Campus of Sun Yat-sen University, Shenzhen, P.R. China; 5 Key Laboratory of Tropical Disease Control, Ministry of Education, Sun Yat-sen University, Guangzhou, P.R. China; University of Cambridge, UNITED KINGDOM OF GREAT BRITAIN AND NORTHERN IRELAND

## Abstract

**Background:**

Dengue is a significant global health threat, transmitted by mosquitoes and influenced by multiple factors. A comprehensive analysis of the impact of these factors on dengue at a global scale is helpful for better understanding and effective control of dengue epidemics.

**Methods:**

This study employed machine learning techniques to develop a global predictive model for forecasting annual dengue cases. A wide range of multi-source features, including historical cases, population, climate, air travel, forest, anemia, vector, serotype and socioeconomic features, were comprehensively considered. The impact of these features was revealed using the SHAP (Shapley Additive Explanations) framework.

**Results:**

The global multi-variable model outperformed the baseline model, indicating the importance of considering multiple factors. Among the multi-source features, historical cases contribute the most, at about 73.63%. Risk factors associated to dengue were identified, including the occurrence of Aedes mosquitoes, changes in the predominant serotype, and the prevalence of anemia. Feature contribution pattern was different between hyperendemic and non-hyperendemic regions. In hyperendemic regions, historical cases and population were found to contribute more significantly, emphasizing the role of population immunity in dengue dynamics.

**Conclusions:**

Dengue is influenced by a wide range of multi-source factors, and prevention and control measures should be specifically designed while taking into account regional differences for effective control of dengue.

## Author summary

Dengue is a serious health issue worldwide, spread by mosquitoes and affected by multiple factors. By studying multi-source factors, including historical cases, population, climate, air travel, forest, anemia, vector, serotype and socioeconomic factors, machine learning models were developed to predict annual dengue cases globally. In addition, impact of the factors on dengue was explored. Historical cases of dengue were found to be the most important factor and the presence of Aedes mosquitoes, changes in the main type of dengue virus and the level of anemia in a population can increase the risk of dengue. Additionally, in areas where dengue is hyperendemic, historical cases and population play a bigger role, showing how important community immunity is in controlling dengue. In conclusion, dengue is affected by many different factors, and strategies to prevent and control it need to be tailored to each region’s specific circumstances to effectively manage dengue outbreaks.

## Introduction

Dengue is an important vector-borne infectious diseases [1]. It is caused by dengue virus, which can be categorized into four serotypes, including DENV-1, DENV-2, DENV-3 and DENV-4 [2]. Dengue is one of the threats to global health with about 390 million infections, 96 million cases and 10,000 deaths per year [3,4]. Nowadays, dengue is expanding to a global scale [5,6], making more and more people under the risk of dengue. However, effective anti-dengue drugs are unavailable [7] and there is no universally applicable vaccine [8,9]. The main measures to control dengue are non-pharmacological interventions, such as vector control, health education and environmental management [10]. The proactive allocation of health resources and implementation of measures is beneficial for the prevention and control of dengue. Therefore, advance prediction of dengue epidemic intensity is important for public health practice.

Timely predictions play a crucial role in disease prevention and control. Actually, numerous studies have concentrated on the short-term forecasting of dengue, encompassing time frames such as the forthcoming week or month [11–13]. Simultaneously, projections concerning long-term dengue trends, covering future periods such as the subsequent three decades or century, have also been conducted [14,15]. However, the temporal scopes forecasted in these studies were either excessively brief or prolonged. Annual forecasts are critically important as they allow policymakers to devise strategies, execute measures, and distribute health resources proactively. In addition, most model-based studies on dengue are typically confined to specific regions [12,16,17], making it unclear whether the same findings can be observed in other regions. Hence, it is crucial to develop a global model that can uncover general patterns of dengue.

Dengue is affected by multiple factors, including the virus itself, the host, the vector and environmental factors. Understanding and considering these various factors are crucial for predicting and managing dengue effectively. Besides of dengue surveillance and weather variables, there are additional factors found associated with dengue [10]. For example, socioeconomic factors have been found correlated to dengue risk, such as accessibility to health care [18], education and GDP [19,20]. It is found that area non-forested variable is significantly associated with dengue in El Salvador [19]. What is more, population and human mobility also contribute an important part in dengue incidence [14]. However, the predictors are based on local contexts and the region-specific models may be precise to the local but it is not conducive to applying the model elsewhere. Besides of that, the vector is definitely helpful for dengue prediction but less considered due to incomplete recording [15,21]. It is found that a status of iron deficiency in the human population might contribute to the vectorial permissiveness to dengue virus, thereby facilitating its spread by mosquitoes [22], indicating the nutritional status of the population may influence dengue epidemic. Moreover, a change of predominant serotype was often associated with severe disease with intense transmission [23,24]. However, this feature is rarely studied and analyzed together with other influencing factors, so it should be considered comprehensively. Combining these factors and putting them into a global context will help to identify essential factors affecting dengue epidemics more generally.

Conventionally, time series models are frequently employed to forecast dengue incidence and regression models utilize historical dengue surveillance data along with relevant weather variables to predict future dengue cases [13,25,26]. However, they may be limited in terms of model assumptions and the number of predictors that can be included. To overcome these limitations, researchers have explored alternative modeling approaches, such as machine learning techniques, to forecast dengue incidence [12,21]. Machine learning models offer more flexibility and can handle complex relationships and a large number of predictors. What is more, the SHapley Additive exPlanation (SHAP) framework has been developed and applied to interpret the contribution of the various predictors of machine learning model [27,28].

Therefore, in this study, multiple factors will be considered systematically and the general risk factors will be figured out in a global scale. Through machine learning technique, multiple variables, including historical cases, population, climate, air travel, forest, anemia, vector, serotype and socioeconomic features, were used to construct global predictive models to predict dengue annual cases. Several machine learning models were carried out and the best was selected to evaluate the performance in each region and further figure out the global and regional feature contribution patterns. The findings may be beneficial for dengue prevention and control.

## Results

### Performance of baseline and multi-variable models

The study employed four distinct machine learning models, analyzing the performance of both baseline and multi-variable models. Baseline models solely utilize historical cases as variables, whereas multi-variable models encompass a comprehensive range of features, including historical cases, population, climate, anemia, air travel, vector, forest, dengue virus serotype and socioeconomic features.

Firstly, the multi-variable models performed better than the baseline models generally. To be specific, based on the four-fold cross validation results (S4 Table), the MSE and RMSE of the four multi-variable models were lower than that of the baseline models. Based on the test set results (Table 1), except MLP model, the MSE and RMSE of the other three multi-variable models were lower than that of the baseline models. Secondly, among the multi-variable models, the random forest model demonstrates better performance than the other three machine learning models (MSE: 0.4623, RMSE: 0.2165, in S4 Table; MSE: 0.4220, RMSE: 0.1781, in Table 1) according to both the four-fold cross validation and test set results. Therefore, the following analysis were based on the random forest model.

**Table 1:** Performance of the models (test set results).

Models	Baseline			Multi-variable		
RMSE	MSE	R^2^	RMSE	MSE	R^2^
Random forest	0.4545	0.2066	0.8176	0.4220	0.1781	0.8428
XGBoost	0.4710	0.2218	0.8042	0.4571	0.2089	0.8156
MLP	0.5164	0.2667	0.7646	0.5317	0.2827	0.7504
SVR	0.6727	0.4525	0.6006	0.6060	0.3672	0.6759

Note: The baseline model only used historical cases features; the multi-variable model used nine categories of features including historical cases, climate, anemia, population, air travel, vector, forest, serotype and socioeconomic factors.

In addition, to test the effect of multi-source features on this model, ablation study was carried out and the change of the performance of the model were displayed in S5 Table. As is displayed in S5 Table, it can be observed that for each type of feature, the mean difference in MSE between the models (MSE of each model can be checked in S2 Data) without and with the feature was positive, indicating that removing any type of feature would weaken the model’s performance (S5 Table). Consequently, integrating these multi-source factors can enhance the model’s performance. Therefore, the multi-variable random forest model was selected to the further analysis.

### Regional predictive performance

**Fig 1:**
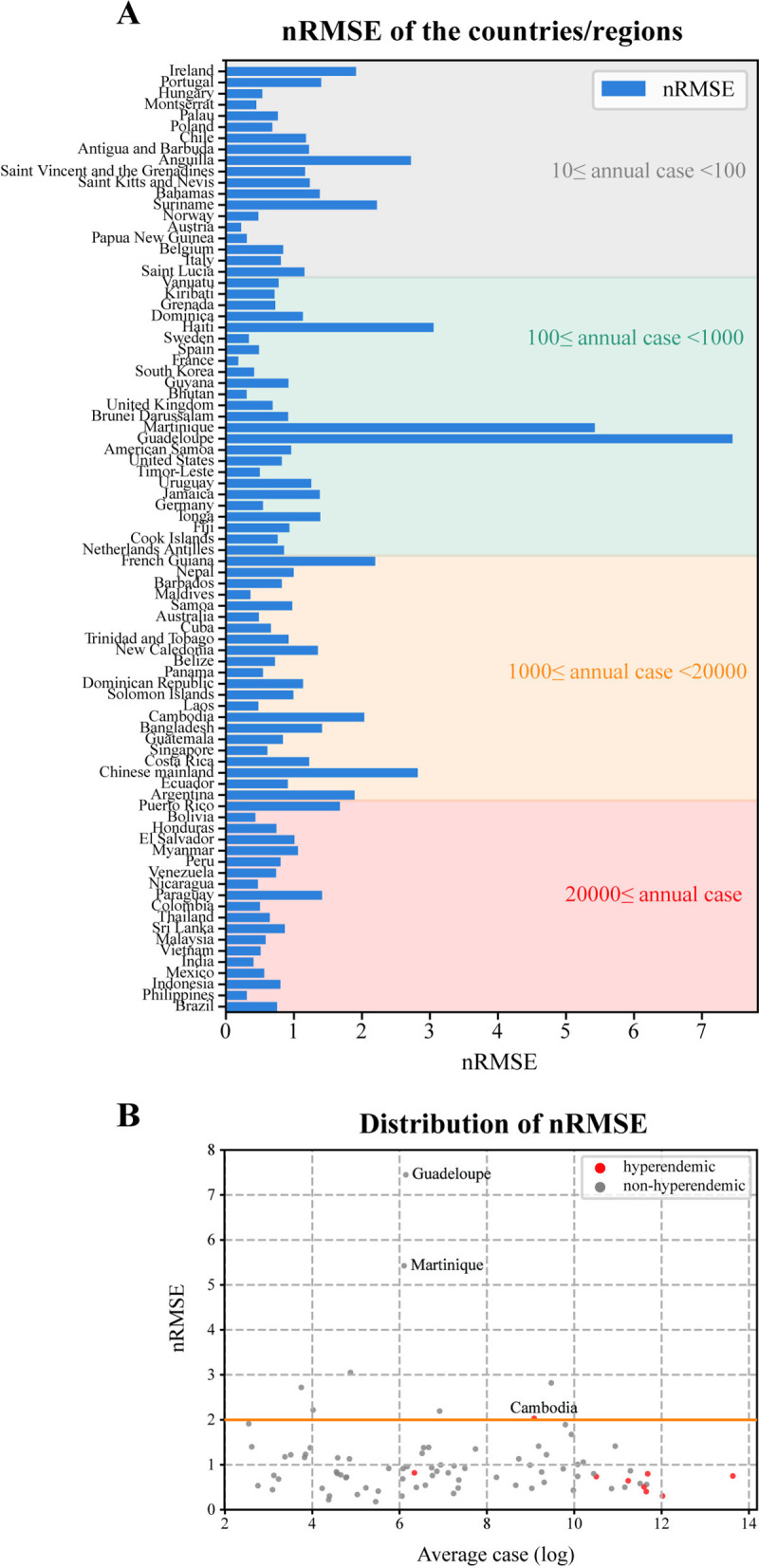
Regional predictive performance. **A)** nRMSE values of the countries/regions. The horizontal axis denotes nRMSE values of the test sets (from 2013 onwards) in the countries/regions. The vertical axis denotes the name of the countries/regions, which were divided into four layers based on their average annual cases and colored with red, orange, light blue and grey, respectively. B) The horizontal axis represents the log-transformed values of annual average case numbers by region. The vertical axis denotes nRMSE values of the test sets (from 2013 onwards) in the regions. Each dot represents a country/region. The results of hyperendemic regions were colored with red. The nRMSE is an indicator for model performance, where less is better.

The developed model had a global scope, encompassing diverse regions with fluctuating annual average dengue cases. To facilitate a direct comparison of predictive efficacy across regions, the normalized root mean squared error (nRMSE) was employed as a metric for assessing regional predictive performance. The nRMSE value was applied to evaluate regions in four distinct layers according to their average annual cases between 10–20,000. In our study, with the threshold of 2, the nRMSE value greater than 2 was considered poor performance and less than 2 was regarded as satisfactory performance, where less than 1 was considered as good performance. By contrast, the nRMSE value more than 3 demonstrated the bad performance. First of all, the nRMSE value was lower than 1 in 65.9% of the regions, and it was below 2 in 90.6% of them (Fig 1). Moreover, Fig 1A delineates the nRMSE values for regions stratified into four distinct layers according to their average annual cases. First, in regions where the average annual case count surpassed 20,000, the nRMSE remained consistently below the threshold of 2. Then, regions with average annual dengue cases ranging from 1,000–20,000 exhibited nRMSE values below 3, with the highest value observed in mainland China, which registered an approximate nRMSE of 2.8. However, in the layer where the average annual cases fell between 100 and 1,000, Guadeloupe, Martinique, and Haiti exhibited poorer predictive performance compared to other regions. In the layer where the average annual cases ranged from 10 to 100, the nRMSE values were all below 3 (Fig 1A). In summary, as demonstrated in Fig 1B, the predictive performance across most regions was notably satisfactory (nRMSE<2 in 90.6% of the regions). It is worth noting that predictive performance for all the regions with average annual cases exceeding 20,000 was deemed satisfactory (nRMSE<2). Significantly, within hyperendemic regions, the nRMSE values remained consistently below 1, with the exception of Cambodia (Fig 1B).

To assess the model’s performance across distinct geographical areas, the representative countries of Europe, Asia and the Americas were selected for specific illustrations. First of all, it can be observed that the model’s performance was optimal in France as evidenced by the lowest nRMSE value of 0.18 (Fig 1A). It is evident that the model was adept at fitting the trend in both the training and test datasets, with a minimal error rate in France (S2A Fig). Then, it was discerned that in Thailand, as an exemplar of an Asian region, the model effectively captured the overall trend within the training dataset, albeit with limited precision (S2B Fig). Conversely, Brazil, representing an American region, exhibited effective trend learning within the training dataset; however, it encountered difficulties in accurately replicating this trend within the test dataset (S2C Fig). Taking into account the variance in case sizes between these regions, the nRMSE value was identified as 0.64 for Thailand, compared to 0.75 for Brazil, thereby indicating a superior performance in Thailand (Fig 1A). Besides of that, to assess and visualize the model’s performance comprehensively, the number of observed cases and predicted cases by the model in countries or regions (cases data available in no less than 10 years) were displayed in S3 Fig. The detailed values of both observed and predicted cases for all regions encompassed within the study are provided in S3 Data.

### Contribution of multi-source features

Moreover, to gain deeper insights into the impact of multi-source features on dengue, the SHAP framework was utilized to explore the contribution of these features in the multi-variable model. First of all, explained variation refers to the portion of the response variable’s variance that can be accounted for by the predictor variables in the model. In this study, the response variable is the number of dengue cases in the next year. Based on the coefficient of determination of the multi-variable random forest model, approximately 84.28% of the variation in the response variable is explained by the included features in the model. This implies that around 15.72% of the variation remains unexplained. Within the explained variation, approximately 85% can be attributed to the original record, while the remaining 15% is associated with the missing record (S4 Fig). The percentage of variation explained by different types of features is detailed in S4 Fig. Notably, historical cases contribute the largest share of the explained variance, accounting for 42.09% (S4 Fig). Population features follow as the second most influential, followed by climate, socioeconomic and air travel features (S4 Fig). This highlights the substantial role of historical cases in predicting future dengue cases, emphasizing the importance of population immunity in dengue epidemics.

**Fig 2:**
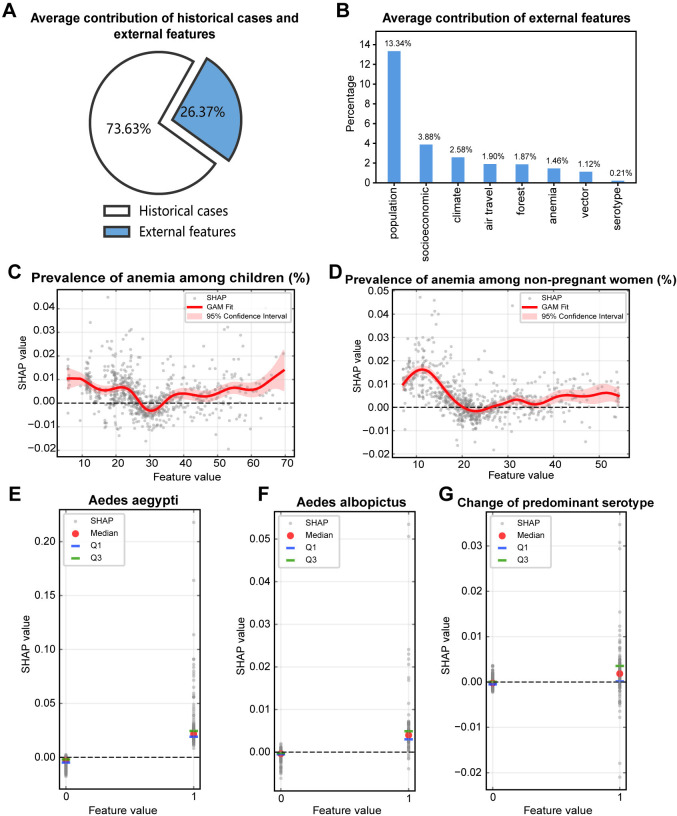
Contribution of multi-source features. (A) demonstrates the relative percentage of the average contribution of the features between historical cases and external features. (B) depicts the mean contribution of each feature across eight categories of external features. (C) and (D) demonstrate the response curves for the prevalence of anemia among children and non-pregnant women, respectively. (E), (F) and (G) illustrate the median and 25th and 75th percentiles of SHAP values of Aedes aegypti, Aedes albopictus and change of predominant serotype, respectively. SHAP value is the contribution of each feature towards the outcome of dengue cases next year. A positive SHAP value indicates that the feature pushes the prediction higher than the average and the opposite is true if it is negative. The response curve is defined as the contribution of the feature to the prediction at different values.

To ensure a fair comparison of the contributions of multi-source features, the relative percentage of the average contribution of the variables was calculated and presented in Fig 2A and 2B. It is evident that historical cases make the most significant contribution, accounting for approximately 73.63% (Fig 2A). Besides of that, population features make the largest average contribution among the external features (Fig 2B), indicating their significant influence on dengue dynamics. Socioeconomic features followed as the second most impactful, followed by climate, air travel, forest, anemia, vector and serotype features (Fig 2B). These findings provide insights into the relative importance of different categories of external features in comprehending dengue epidemics.

To further validate the impact of the variables on dengue, response curves for continuous variables were generated to explore their delicate relationship with dengue, as displayed in Figs 2C, 2D and S5–S8. Meanwhile, the median and 25th and 75th percentiles of SHAP values of binary variables were obtained to demonstrate their influence on dengue, as displayed in Fig 2E–G. In general, the relationships between the continuous features and SHAP values exhibit nonlinearity, highlighting the complex interactions between multi-source features and dengue. For example, for the feature of precipitation, the response curve reveals that when it is below 2000mm, the impact is positive, while above 2000mm, the impact turns negative (S6 Fig). Similar results can be found to the features of diurnal temperature range, rain days, and cloud cover.

It is worthy to mention three categories of features, including anemia, vector, and serotype features which have been relatively less studied. Therefore, their impact on dengue will be elaborated specifically on Fig 2C‒G. Firstly, regarding to the anemia features, the response curve of prevalence of anemia among children typically remained above the 0 line, except for the prevalence around 30% (Fig 2C). Similarly, for the prevalence of anemia among non-pregnant women, the curve was mostly above 0, except for some instances around 23% (Fig 2D). The findings suggest that prevalence of anemia may be a risk factor for dengue generally. Secondly, as to the vector features, when their values was equal to 1, which corresponds to the presence of Aedes vectors, the median SHAP value was greater than 0 (Fig 2E and 2F), indicating that the occurrence of Aedes aegypti and Aedes albopictus are risk factors for dengue. Lastly, when a change in the predominant serotype was observed, the median SHAP value exceeded 0 (Fig 2G), signifying that alterations in the predominant serotype are associated with an increase in dengue cases. In conclusion, the findings illustrate that the presence of Aedes mosquitoes, shifts in the predominant serotype, and the prevalence of anemia amongst both children and non-pregnant women are likely risk factors for dengue. These factors merit consideration in the formulation and execution of strategies for the prevention and control of dengue.

### Regional pattern of feature contribution

In order to understand the impact of multi-source features on dengue, the regional patterns of feature contribution were examined, taking into account hyperendemic and non-hyperendemic regions. Hyperendemic regions are defined as regions where all four serotypes of dengue circulate locally above certain thresholds [29].

**Fig 3:**
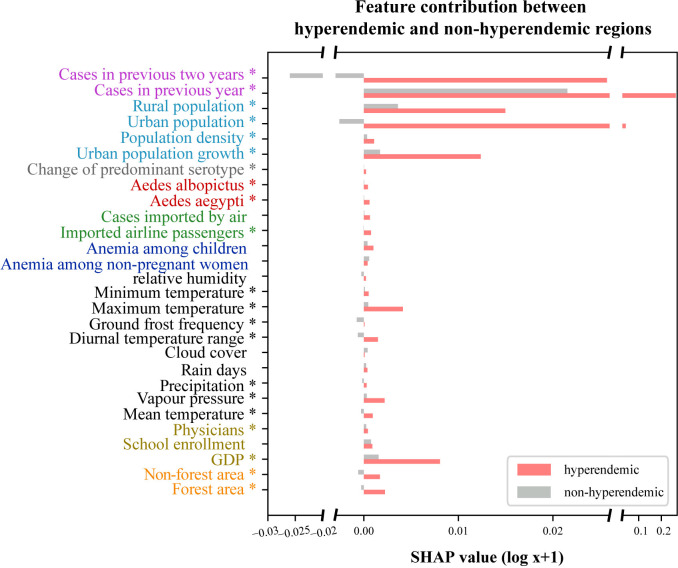
Comparison of feature contribution between hyperendemic and non-hyperendemic regions. The multi-source different categories of features are represented with different colored fonts. Red and grey bars represent results of hyperendemic and non-hyperendemic regions, respectively. Asterisks indicate statistically significant results (p < 0.05).

Comparison of regional pattern of feature contribution was conducted using Mann-Whitney test based on the SHAP values of each feature between hyperendemic and non-hyperendemic regions, as depicted in Fig 3. If all the variables within a category of features are significantly different, then the category of feature is considered to be different between hyperendemic and non-hyperendemic regions. It was observed that overall, there were five categories of features, including historical cases, population, forest, vector and serotype, where all features exhibit significant differences between the two types of regions (Fig 3).

In particular, historical cases and population contribute the most to all features in both hyperendemic and non-hyperendemic regions but these two categories of features were found significantly different between them (Fig 3). Historical cases and population contribute more in hyperendemic regions compared to non-hyperendemic regions in general. Cases in the previous two years and urban population had a negative impact in non-hyperendemic regions compared to the hyperendemic ones (Fig 3). Consequently, it can be concluded that historical cases and population, which are indicative of population immunity, play an essential role in shaping dengue epidemics within hyperendemic regions.

Moreover, vector and serotype features also exhibit significant differences between the hyperendemic and non-hyperendemic regions (Fig 3). Climate, socioeconomic, air travel and anemia features were not found to be significantly different between them (Fig 3). Besides, it can be noted that urban population growth, GDP, and maximum temperature have a greater effect in hyperendemic regions than non-hyperendemic regions (Fig 3). Based on these findings, it may be inferred that the historical incidence and demographic characteristics, which indicate the immunity levels of the population, are fundamental in influencing dengue epidemics within hyperendemic regions. Additionally, occurrence of Aedes mosquitoes and predominant serotype switching have a greater impact on dengue epidemics in hyperendemic regions. This underscores the critical importance of implementing interventions that target the host, the virus and the vector to effectively control dengue in these regions.

Besides of that, the pattern of feature contributions between regions in the Americas and Asia was explored and compared by the same method. It can be observed that historical cases and anemia features were significantly different between regions in the Americas and Asia (S9 Fig).

## Discussion

In this study, a global predictive model was developed utilizing comprehensive multi-source features to anticipate annual dengue cases. The impact of these features was evaluated through the SHAP framework. It was found that historical cases were the biggest contributors and the inclusion of multi-source external features improved the performance of the model. Nevertheless, approximately 15% of the outcome variance remained unexplained. Notably, the presence of Aedes mosquitoes, changes in the predominant serotype, and the prevalence of anemia were identified as risk factors for dengue. Historical cases and population played a more significant role in dengue epidemics in hyperendemic regions, highlighting the importance of population immunity in those regions. Moreover, presence of Aedes mosquitoes and predominant serotype switching have a greater impact on dengue epidemics in hyperendemic regions than non-hyperendemic regions. This underscores the need to implement comprehensive measures targeting the multi-source factors about the host, the virus and the vector to effectively control dengue.

Dengue is influenced by a range of factors, including the environment, the host and the virus itself. However, current research on risk factors for dengue is often limited to specific regions or focuses on specific factors [18–20], leading to a lack of comprehensive studies. It is crucial to systematically explore the impact of multiple factors on dengue at a global scale. In this study, we aimed to address this research gap by comprehensively considering multiple factors known to be associated with dengue using publicly available data. These factors include historical cases, climate, anemia, population, air travel, vector, forest, serotype and socioeconomic factors. By considering all these factors, a comprehensive understanding of their influence on dengue dynamics can be achieved. Apart from examining multiple factors, this study also encompassed over 100 regions worldwide to develop the predictive model. Despite variations in predictive performance across different regions due to regional heterogeneity, compared to the baseline model, based on the lower RMSE, the model demonstrated good predictive capability across most regions with varying disease burdens. This promising performance provides inspiration and suggests that the global model developed in this study has the potential to serve as a valuable substitute for estimation of future dengue cases, particularly when there are no local models in resource-deficient areas. Nevertheless, certain regions such as Guadeloupe and Martinique exhibit inferior performance relative to other regions, which may be attributable to a greater extent of missing feature data pertaining to these regions. Furthermore, the unmeasured confounders and inherent epidemiological differences may contribute to the heterogeneous predictive performance observed across regions. Therefore, to enhance regional predictive capability, it is advisable to fortify local surveillance systems to ensure comprehensive data collection regarding cases and risk factors, and to investigate the intrinsic epidemiological variations specific to each region.

Analysis of multi-source features revealed that historical cases represent the most significant contributor to dengue, a result analogous to findings related to influenza [30]. This underscores the importance of population immunity in shaping future infection magnitudes, despite the differing scenarios among various pathogens. Furthermore, historical cases exert a more pronounced effect in hyperendemic regions compared to non-hyperendemic regions, emphasizing the pivotal role of population immunity in these areas. Actually, it has mentioned that independent of climate variability over the years, population susceptibility cycles preserve to some extent [31]. Moreover, it is also reported that antibody boosting and waning in the highly exposed individuals play a role in driving dengue epidemic dynamics [32]. Therefore, the importance of historical cases aligns with the significance of the population’s history of dengue infection by different serotypes [32,33], particularly in hyperendemic regions where all four serotypes are circulating simultaneously [34]. Furthermore, the negative impact of cases in previous two years in non-hyperendemic regions may also be associated to the interplay of population susceptibility cycles and infection history, which requires further verification, particularly the dynamics of seroprevalence in those regions.

Furthermore, based on the response curves and SHAP values, several risk factors for dengue were identified, including the presence of Aedes mosquitoes, the change of predominant serotype, and the prevalence of anemia. The presence of Aedes mosquitoes, whether *Aedes albopictus* or *Aedes aegypti*, was confirmed as an essential contributor to dengue transmission in this study. In addition, the change of predominant serotype was found to be associated with intense transmission or large-scale outbreaks of dengue [23,24]. However, previous studies rarely considered it in conjunction with other risk factors such as climate and air travel. This study, by considering multiple influencing factors, demonstrated that the change of predominant serotype is indeed a risk factor for dengue. This highlights the importance of understanding the distribution and surveillance of serotypes to effectively prevent and control dengue outbreaks. Moreover, the prevalence of anemia was taken into account for the first time in exploring its impact on dengue in this study. Previous research suggested that the presence of iron deficiency in the human population might contribute to vectorial permissiveness to the dengue virus, thereby facilitating its spread by mosquitoes [22]. The results of this study align with this inference, as the response curves for both the prevalence of anemia among children and non-pregnant women show that higher prevalence is associated with an increase in the number of dengue cases. This may suggest that dengue prevention and control efforts can be initiated by improving the nutritional status of the population, particularly by reducing the prevalence of anemia. Additionally, another possibility is that the prevalence of dengue and anemia affects each other, and the causal relationship here needs further research to confirm. Furthermore, it has been observed that the influence of climate on dengue is considerable, corresponding to previous studies [35,36], and generally demonstrates a non-linear nature. Air travel exacerbates the spread of dengue epidemics, as previously documented in the studies mentioned [37,38]. The expansion of dengue epidemics is shaped by both social and natural environmental determinants [19,20]. Overall, these findings underscore the importance of considering and addressing multiple risk factors, including the presence of Aedes mosquitoes, changes in the predominant serotype, and the prevalence of anemia, in comprehensive dengue prevention and control strategies.

Besides, an interesting observation was made regarding the different feature contribution patterns between hyperendemic and non-hyperendemic regions. In hyperendemic regions, historical cases and population were found to contribute more significantly to dengue epidemics. This suggests that the status of population immunity plays a crucial role in the dynamics of dengue in these regions, as all four serotypes of the virus circulate locally [29]. What is more, presence of Aedes mosquitoes and predominant serotype switching have a greater impact on dengue epidemics in hyperendemic regions, demonstrating the need to implement surveillance on the changes in dengue serotypes because it is a risk factor to dengue. In contrast, the contribution pattern of climate, socioeconomic, air travel, and anemia factors were not significantly different between non-hyperendemic and hyperendemic regions. The discovery of regional heterogeneity in feature contribution patterns informs the implementation of prevention and control measures in a focused and tailored manner in different endemic regions. In hyperendemic regions, although comprehensive approaches should be adopted to effectively control dengue, measures focusing on population immunity are crucial.

However, it is important to acknowledge certain limitations in this study. Firstly, bias exists in the surveillance of both dengue cases and associated risk factors. Dengue cases considered in this study are based on reported cases only, which may underestimate the true scale of the epidemic as asymptomatic cases were not included. And dengue cases data of different serotypes are unavailable. In addition, due to the absence of comprehensive systematic monitoring, there are missingness of the features data. To reduce the impact of surveillance bias during data analysis, analytical adjustments and transparency were focused on in this study. For dengue cases, we reported the cases data source transparently. For the features data, we utilized the missing indicator method to adjust missing data and performed the sensitivity testing. Therefore, bias in surveillance may not be completely avoided, but the impact can be reduced to some extent. To improve and avoid bias in surveillance, during surveillance, representative sampling and consistent detection methods across all populations should be ensured in the future. Key strategies include standardized case definitions, broad testing coverage and continuous monitoring. Furthermore, in this study, vector and serotype features were represented as binary variables due to inadequacies in surveillance. A value of 0 was assigned to indicate absence or an unknown status. Consequently, while this representation sufficed for the purposes of model training, there remains a potential risk of underestimating these features. Secondly, although the study aimed to include a comprehensive set of multi-source factors, approximately 15% of the variance remained unexplained. This suggests that there may be more nuanced factors that are closely associated with dengue transmission and epidemics that were not captured in this analysis. For instance, a recent study found that sea surface temperature anomalies and a climate index for the Indian Ocean basin can predict the magnitude of dengue outbreaks [39], which were not taken into account in this study. Moreover, features concerning policy and vector control program changes were not included in the model due to the lack of relevant data. This limitation may contribute to the model’s inability to accurately forecast future scenarios in certain regions. Future research should delve deeper into identifying and incorporating more factors to improve our understanding of dengue dynamics. Furthermore, focusing on a comprehensive consideration of the relationship between multi-source factors and dengue, the specific causal relationship between each factor and dengue is difficult to be covered in this study. Lastly, because the model developed in this study has a global scope, it is important to acknowledge the need for regional application considering the heterogeneity of different regions. By incorporating region-specific information and adjusting the model accordingly, more accurate and contextually relevant predictions can be obtained. Additionally, given the big contribution of historical cases to model performance, the model cannot predict the timing of an introductory dengue outbreak, rather it predicts the next epidemic when dengue is already endemic. Furthermore, the differentiation between local and imported dengue cases was not implemented within the model due to the unavailability of requisite data. We anticipate the acquisition of more comprehensive data to facilitate the development of distinct predictive models for local and imported dengue cases, thereby enhancing precision.

In summary, a global-scale predictive model incorporating multiple factors was developed to forecast dengue cases one year in advance. This model allowed us to uncover the impact of various environmental, host, and viral factors, revealing diverse feature patterns across regions. These findings enhance our understanding of the complex relationship between multiple factors and dengue, providing valuable insights for the prevention and control of this disease. By leveraging these findings, we can work towards more effective strategies to mitigate the impact of dengue and protect public health.

## Methods and materials

### Annual case data collection and processing

Annual dengue reported cases data were collected from WHO dengue data application (https://ntdhq.shinyapps.io/dengue5/). Dengue cases include clinically suspected and laboratory confirmed cases. These cases data cover 120 countries or regions from 1990 to 2018. First of all, those with no case record were removed and finally 104 countries or regions (listed in S1 Table) with cases reported (case≥1) were taken into analysis. There were 1776 annual dengue cases samples in total. The scale of recording for each sample was the total number of reported dengue cases per region per year. Subsequently, as the samples of dengue cases is a long-tailed distribution (S1A Fig), box-cox transformation was performed to handle this and the parameter of lambda was set as 0.09, then the distribution of the transformed cases is near normal (S1B Fig).

### Multi-source features representation and processing

Multi-source features associated with dengue epidemic were collected. Totally, there are nine categories of features, including historical cases, climate, anemia, population, air travel, vector, forest, serotype and socioeconomic factors. Historical cases represent the immune status of the population. Except for historical cases, the other eight categories of features defined as external features. To make predictions about dengue cases in the coming year, the previous year’s features were used to construct the model. In addition, there are missing values for the features. Fortunately, missing indicator methods have been applied to many medical studies and imputation with missing indicator method have used in studies [40,41]. To address the issue of missing values and assess their impact, mean value imputation alongside a missing indicator was utilized to manage the feature missingness in this study. The approach involved two steps: impute missing values with the mean, then add a binary indicator for each original variable (1 for missing, 0 for present). Consequently, the feature set consisted of both the original and the missing indicator components. Furthermore, a sensitivity analysis was conducted concurrently to compare the multiple imputation method, in which the estimator used was KNeighborsRegressor, with the maximum number of iterations set at 100, for addressing the missing values. The total nine categories of the features and the variables included in each category were described in detail in the followings.

#### Historical cases.

Historical cases data were collected from WHO dengue data application as described above. The effect of historical cases within a cycle was taken into account. Given that the common cycle for dengue ranges from 2 to 5 years [42,43], the shortest interval was considered. Therefore, *cases in the previous year* and *cases in the previous two years* in a given region were used to represent its historical cases. In other words, the features representing historical cases consisted of two variables: *cases in the previous year* and *cases in the previous two years.*

#### Climate.

The climate features were composed of *diurnal temperature range*, *cloud cover*, *ground forst frequency*, *precipitation*, *mean temperature*, *maximum temperature*, *minimum temperature*, *rain days*, *vapour pressure* and *relative humidity*. The annual mean values of the former nine variables at the regional or national level were collected from the CRU CY4.05 dataset [44], and relative humidity was calculated based on vapour pressure and mean temperature through the empirical formula recommended by Emanuel [45].

#### Population.

The annual population data of the countries or regions were collected from World Bank (https://databank.worldbank.org/reports.aspx?source=World-Development-Indicators). Population features are composed of four variables, including *population density*, *urban population*, *rural population*, and *urban population growth*. The variables were assigned the value retrieved from the database.

#### Socioeconomic feature.

The annual socioeconomic data of the countries or regions were also collected from the World Bank (https://databank.worldbank.org/reports.aspx?source=World-Development-Indicators). The socioeconomic features include GDP per capita (*GDP*), physicians per 1,000 people (*physicians*), and secondary school enrollment (*school enrollment*). The variables were assigned the value retrieved from the database.

#### Air travel.

Airline passenger flow served as a representation of human mobility. The data were collected from the Official Aviation Guide (https://analytics.oag.com/analyser-client/home). Considering the influence of travelers entering from abroad on the transmission of dengue, the annual data for *imported airline passengers* and *cases imported by air* were taken into account. *Imported airline passengers* was defined as the total number of inbound air passengers from foreign locations. *Cases imported by air* was estimated based on the incidence rate of dengue in the place of departure. (*Cases imported by air* =* Imported airline passengers*×dengue incidence rate of the place of departure). The two variables were employed to represent the impact of travelling. As the annual airline passenger flow data are only available from 2011 to 2019, data for the years before 2011 were calculated based on the increase in passengers relative to 2011 as estimated by the World Bank [14].

#### Anemia.

The annual prevalence of anemia of the countries or regions were collected from the World Bank (https://databank.worldbank.org/reports.aspx?source=World-Development-Indicators). It is found that the status of iron deficiency in the human population might contribute to the vectorial permissiveness to dengue virus, thereby facilitating its spread by mosquitoes [22]. To explore the impact of anemia status of the population on dengue, the *prevalence of anemia among non-pregnant women* (% of women ages 15–49) and *prevalence of anemia among children* (% of children ages 6–59 months) were used to represent the anemia features. The variables were assigned the value retrieved from the database.

#### Forest.

*Coverage of forested* and *non-forested areas*, which were obtained from World Bank (https://databank.worldbank.org/reports.aspx?source=World-Development-Indicators), were used to represent the forest features. The variables were assigned the value retrieved from the database.

#### Vector.

The presences data of vector were obtained from the database [46]. The vector features include presences of *Aedes albopictus* and that of *Aedes aegypti*. For specific region in specific year, 1 represents the presence and 0 represents no presence or unclear. Therefore, the two vector variables were all filled with 0 or 1.

#### Dengue virus serotype.

*Change of predominant serotype* was considered and represented virus genetic feature impact on dengue cases. The envelope (E) gene sequences of dengue virus of the four serotypes were collected from Dengue virus database of National Center for Biotechnology Information (NCBI) [47] for different regions in different years. For the specific region in the year, predominant serotype was defined as the one with the highest number of all the four serotypes. Compared to the previous year or the previous two years (if no sequence was available in the previous year), serotype feature was indicated by 1 if the predominant serotype had changed, and 0 otherwise. Thus, this variable was a binary variable, filled with 0 or 1.

### Model construction and evaluation

#### Baseline model and multi-variable model.

The baseline models were constructed using historical cases only, while the multi-variable models were developed utilizing the total nine categories of features (comprising 28 variables), including historical cases, climate, anemia, population, air travel, vector, forest, serotype and socioeconomic factors. In order to have a higher performance, a series of machine learning models were employed, encompassing Support Vector Regression (SVR), XGBoost, Random Forest and Multi-Layer Perceptron (MLP). The SVR and Random Forest models were implemented in Python 3.9 using the sklearn package. The XGBoost model was constructed utilizing the xgboost package, while the MLP model was developed through the torch package. The hyperparameters of these models are detailed in S2 Table. The model demonstrating superior performance (least MSE) was chosen for subsequent analysis. To mitigate the influence of the year factor [48], the training and testing datasets were randomly sampled across the years, followed by the execution of a four-fold cross-validation process. The outcome of the models was the number of dengue cases in next year.

#### A sensitivity analysis of methods for imputing missing values.

In order to compare the efficacy of the missing indicator method against multiple imputation techniques in addressing missing data (refer to the section on multi-source representation and processing), a four-fold cross-validation was implemented across baseline and multi-variable models for each imputation method. Ultimately, the missing indicator method was selected, given its enhanced performance within the model (S3 Table presents the results of the multiple imputation method; S4 Table presents the results of the missing indicator method).

#### Model evaluation.

The models were evaluated using mean squared error (MSE), root mean squared error (RMSE) and the coefficient of determination (R^2^). The mean squared error measures the average squared difference between the estimated values and the observed value. The root mean squared error is computed as the square root of the average of the squared variances between the predicted and observed values. The coefficient of determination represents the proportion of the variation in the dependent variable that can be explained by the independent variables.

#### Ablation study.

To determine the contribution of each feature category to model performance, all combinations of the nine feature categories were thoroughly explored [49,50]. The impact of a specific feature category on the model was assessed by evaluating the variation in model performance between combinations that included and excluded that specific feature category. The change of MSE metrics were used to evaluated the impact of the feature categories through four-fold cross validation. For instance, when considering the climate category, we firstly assessed the model’s performance by employing a combination of climate, population, and socioeconomic features (as one of the combinations). Subsequently, we evaluated the model’s performance with only the population and socioeconomic features, and determined the difference in performance between these two model setups. Ultimately, within the total of nine feature categories, we comprehensively examined all combinations including climate features and their corresponding combinations lacking climate features to ascertain the differential impact on model performance attributable to climate features. (details of the feature combinations and the corresponding model performance results can be found in the S2 Data). The remaining eight categories of features were tested in the same way.

### Regional predictive performance

Furthermore, in order to assess the predictive efficacy of the model for subsequent periods, data preceding and including the year 2013 were utilized as the training set, while data from 2013 onwards were designated as the test set, aligning with methodologies adopted in prior research studies. [16,51,52]. The multi-variable random forest, which demonstrated superior performance among the models previously described, was employed for this evaluation. The hyperparameter configuration employed was consistent with that of the aforementioned random forest model (S2 Table).

Afterwards, to gain insights into the predictive performance of the model across diverse regions, the best-trained multi-variable random forest model was employed to predict regional cases. However, the disparate magnitudes of dengue epidemics across regions present a challenge in facilitating direct comparisons. To overcome this challenge, the metric used to evaluate the predictive performance on the test set within each region individually was the root mean squared error normalized by the regional average number of cases (nRMSE). This metric, referencing the coefficient of variation [53] and the previous study [54], takes into account both the observed cases and the predicted cases, providing a standardized assessment of predictive performance that considers the heterogeneous scales of dengue epidemics across different regions. The normalized RMSE was calculated as:


$$\begin{array}{c} nRMSE=\frac{RMSE}{AVERAGE} \end{array}$$ (1)

The RMSE denotes root mean square error, measuring the average magnitude of the difference between the predicted values and the observed values. AVERAGE denotes the average number of cases per year in each region. Therefore, a lower value of *nRMSE* indicates superior predictive performance.

To assess the comprehensive predictive performance across different regions, a scatter plot was developed utilizing the log-transformed values of annual average case numbers and nRMSE values specific to each region. Furthermore, given the varying magnitude of dengue cases worldwide, regions were categorized into four tiers(10 ≤ case<100, 100 ≤ case<1000, 1000 ≤ case<20000, 20000 ≤ case) based on the annual average case numbers, with the aim of testing and comparing the predictive performance of the model in regions with different epidemic sizes. Detailed nRMSE values were then presented to analyze the predictive performance across regions.

### Feature interpretation

The SHAP (SHapley Additive exPlanations) framework is a unified framework for explaining the output of any machine learning model. It provides a unified way to interpret the contributions of individual features in the prediction of a model. SHAP values are based on cooperative game theory and use the concept of Shapley values to allocate the contribution of each feature to the prediction. The mathematical expression of SHAP value is as:


$$\begin{array}{c} g\left(z^{\prime }\right)=\Phi_{0}+\sum_{j=1}^{N}\Phi_{j} \end{array}$$ (2)

$g\left(z^{\prime }\right)$ denotes the explanatory model. N represents the number of input features. $\Phi_{j}$ denotes the SHAP value of each feature and $\Phi_{0}$ is a constant. For each prediction sample, the model $g\left(z^{\prime }\right)$ calculates a prediction value and the SHAP value represents the contribution of each feature in the sample. Thus, the meaning of SHAP value should be interpreted as the contribution of each feature towards the outcome of dengue cases in the next year in this study. A positive SHAP value indicates that the feature pushes the prediction higher than the average and the opposite is true if it is negative.

#### Proportion of variation explained by multi-source features.

By obtaining the SHAP values for each feature and considering the coefficient of determination of the best-trained random forest model, the percentage of variation explained by each category of features was obtained. The proportion of variation explained by both original and missing records of multi-source features in the model were measured, according to the value of coefficient of determination. This was accomplished by calculating the proportion of SHAP values attributed to each category of feature, providing insights into the relative contribution of different feature categories.

#### Overall contribution of multi-source features.

To obtain a comprehensive understanding of the impact of each feature, a horizontal comparison of the contributions of multi-source features was conducted. Firstly, to get the horizontal comparison of the contributions of historical cases and the external feature (eight categories) to the outcome of dengue cases in the model (the total contribution of them was regarded as 100%), based on the SHAP values of each feature, the average percentage of them were calculated. Then, the eight categories among the external features were measured individually for specific comparison. This analysis aimed to provide the horizontal comparison of the contributions of each feature category to the outcome of dengue cases in the next year in the best performance random forest model.

#### Response curves of the features.

For a more detailed elucidation of the impact of each feature variable, the response curves of the continuous features were generated using a generalized additive model (GAM) based on the feature values and their corresponding SHAP values. Each feature’s response curve was fitted individually to analyze its relationship with the predicted outcome. By employing GAM, a flexible non-linear modeling approach, the response curves provided a visual representation of how each feature’s value influenced the model’s predictions. As for binary variables, the median and 25th (Q1) and 75th (Q3) percentiles were highlighted to visually demonstrate the influence of each feature’s value on the model’s predictions.

#### Regional comparison.

Furthermore, the variations in the pattern of feature contributions between hyperendemic and non-hyperendemic regions were examined. Hyperendemic regions are defined as regions where all four serotypes of dengue circulate locally above certain thresholds [29], including Thailand, Brazil, Venezuela, Cambodia, India, Indonesia, Vietnam, the Philippines and USA. This analysis entailed comparing the SHAP values of each feature between hyperendemic and non-hyperendemic regions using the Mann-Whitney test. It aimed to evaluate the significance of each feature within their respective groups to ascertain the discrepancies in feature importance across the two types of regions. Besides of that, to explore the variations in the pattern of feature contributions between regions in the Americas and Asia, the same evaluation was performed simultaneously.

## Supporting information

S1 Table Regions included in the study and the years with cases data available. (DOCX)

S2 Table Hyperparameters of the machine learning models. (DOCX)

S3 Table Performance of the models through multiple imputation method (Four folds cross validation results). (DOCX)

S4 Table Performance of the models through missing indicator method (Four folds cross validation results). (DOCX)

S5 Table The impact of removing various features on model performance (evaluated by mean MSE difference). (DOCX)

S1 Fig Distribution of the cases data. (PDF)

S2 Fig The number of true cases and cases predicted by the model in three representative regions. (PDF)

S3 Fig The number of true cases and cases predicted by the model in regions (data available in no less than 10 years). (PDF)

S4 Fig Variation explained by all the categories of the features. (PDF)

S5 Fig Response curves of air travel, forest and historical cases features. (PDF)

S6 Fig Response curves of climate features. (PDF)

S7 Fig Response curves of socioeconomic features. (PDF)

S8 Fig Response curves of population features. (PDF)

S9 Fig Comparison of feature contribution between regions in Asia and the Americas. (PDF)

S1 Data Feature data and missingness for the regions.csv. (CSV)

S2 Data Ablation study results.xlsx (XLSX)

S3 Data Regional observed cases and predicted cases.xlsx. (XLSX)
