# Supplementary material for: Annual global dengue dynamics are related to multi-source factors revealed by a machine learning prediction analysis
Source: PLoS Negl Trop Dis. 2025 Jun 25;19(6):e0013232. doi: 10.1371/journal.pntd.0013232 (PMC12221171; doi:10.1371/journal.pntd.0013232)
Supplement: S1 Table — (DOCX) [file pntd.0013232.s001.docx]

**S1 Table. Regions included in the study and the years with cases data available.**

| **Number** | **Region** | **Years (cases data available)** |
| --- | --- | --- |
| 1 | American Samoa | 1994, 1995, 1996, 1998, 1999, 2001, 2002, 2006, 2007, 2008, 2009, 2010, 2015 |
| 2 | Anguilla | 1990, 1995, 1996, 1999, 2000, 2001, 2002, 2003, 2008, 2010, 2011, 2012, 2013, 2014, 2015, 2016, 2017 |
| 3 | Antigua and Barbuda | 1990, 1991, 1995, 1996, 1997, 1998, 1999, 2000, 2001, 2002, 2008, 2010, 2011, 2012, 2013, 2014, 2015, 2016, 2017, 2018 |
| 4 | Argentina | 1998, 1999, 2000, 2001, 2002, 2003, 2004, 2005, 2006, 2007, 2008, 2009, 2010, 2011, 2012, 2013, 2014, 2015, 2016, 2017, 2018 |
| 5 | Australia | 1991, 1992, 1993, 1994, 1995, 1996, 1997, 1998, 1999, 2000, 2001, 2002, 2003, 2004, 2005, 2006, 2007, 2008, 2009, 2010, 2011, 2012, 2013, 2014, 2015, 2016, 2017 |
| 6 | Austria | 2010, 2012, 2013, 2014, 2015, 2016, 2017 |
| 7 | Bahamas | 1990, 1995, 1998, 2003, 2004, 2006, 2008, 2010, 2011, 2012, 2013, 2014, 2015, 2016, 2017, 2018 |
| 8 | Bangladesh | 1999, 2000, 2001, 2002, 2003, 2004, 2005, 2006, 2007, 2008, 2009, 2010, 2011, 2012, 2013, 2014, 2015, 2016, 2017 |
| 9 | Barbados | 1990, 1991, 1992, 1993, 1994, 1995, 1996, 1997, 1998, 1999, 2000, 2001, 2002, 2003, 2004, 2005, 2006, 2008, 2009, 2010, 2011, 2012, 2013, 2014, 2015, 2016, 2017 |
| 10 | Belgium | 2013, 2014 |
| 11 | Belize | 1990, 1995, 1997, 1998, 1999, 2000, 2001, 2002, 2004, 2005, 2006, 2007, 2008, 2009, 2010, 2011, 2012, 2013, 2014, 2015, 2016, 2017, 2018 |
| 12 | Bermuda | 1999, 2005, 2006, 2010, 2011, 2013, 2014, 2016 |
| 13 | Bhutan | 2004, 2005, 2006, 2007, 2008, 2009, 2010, 2011, 2012, 2013, 2016, 2017 |
| 14 | Bolivia | 1990, 1991, 1992, 1993, 1994, 1995, 1996, 1997, 1998, 1999, 2000, 2001, 2002, 2003, 2004, 2005, 2006, 2007, 2008, 2009, 2010, 2011, 2012, 2013, 2014, 2015, 2016, 2017, 2018 |
| 15 | Brazil | 1990, 1991, 1992, 1993, 1994, 1995, 1996, 1997, 1998, 1999, 2000, 2001, 2002, 2003, 2004, 2005, 2006, 2007, 2008, 2009, 2010, 2011, 2012, 2013, 2014, 2015, 2016, 2017, 2018 |
| 16 | Brunei Darussalam | 1995, 1998, 1999, 2004, 2005, 2006, 2007, 2008, 2009, 2010, 2011, 2014 |
| 17 | Cambodia | 1990, 1991, 1992, 1993, 1994, 1995, 1996, 1997, 1998, 1999, 2000, 2001, 2002, 2003, 2004, 2005, 2006, 2007, 2008, 2009, 2010, 2011, 2012, 2013, 2014, 2015, 2016, 2017 |
| 18 | Chile | 2002, 2006, 2007, 2008, 2009, 2010, 2011, 2012, 2013, 2014, 2015, 2016, 2017, 2018 |
| 19 | Chinese mainland | 1990, 1991, 1992, 1993, 1994, 1995, 1996, 1997, 1998, 1999, 2000, 2001, 2002, 2003, 2004, 2005, 2006, 2007, 2008, 2009, 2010, 2011, 2014, 2015, 2016, 2017 |
| 20 | Colombia | 1990, 1991, 1992, 1993, 1994, 1995, 1996, 1997, 1998, 1999, 2000, 2001, 2002, 2003, 2004, 2005, 2006, 2007, 2008, 2009, 2010, 2011, 2012, 2013, 2014, 2015, 2016, 2017, 2018 |
| 21 | Cook Islands | 1990, 1991, 1992, 1994, 1995, 1996, 1997, 2001, 2002, 2003, 2004, 2006, 2007, 2008, 2009, 2014, 2015 |
| 22 | Costa Rica | 1993, 1994, 1995, 1996, 1997, 1998, 1999, 2000, 2001, 2002, 2003, 2004, 2005, 2006, 2007, 2008, 2009, 2010, 2011, 2012, 2013, 2014, 2015, 2016, 2017, 2018 |
| 23 | Cuba | 1997, 2000, 2001, 2002, 2005, 2007, 2009, 2013, 2014, 2015, 2016, 2017, 2018 |
| 24 | Dominica | 1990, 1991, 1993, 1994, 1995, 1996, 1998, 1999, 2000, 2001, 2004, 2005, 2006, 2007, 2008, 2009, 2010, 2011, 2012, 2013, 2014, 2015, 2016, 2017, 2018 |
| 25 | Dominican Republic | 1990, 1991, 1992, 1993, 1994, 1995, 1996, 1997, 1998, 1999, 2000, 2001, 2002, 2003, 2004, 2005, 2006, 2007, 2008, 2009, 2010, 2011, 2012, 2013, 2014, 2015, 2016, 2017, 2018 |
| 26 | Ecuador | 1990, 1991, 1992, 1993, 1994, 1995, 1996, 1997, 1998, 1999, 2000, 2001, 2002, 2003, 2004, 2005, 2006, 2007, 2008, 2009, 2010, 2011, 2012, 2013, 2014, 2015, 2016, 2017, 2018 |
| 27 | El Salvador | 1990, 1991, 1992, 1993, 1994, 1995, 1996, 1997, 1998, 1999, 2000, 2001, 2002, 2003, 2004, 2005, 2006, 2007, 2008, 2009, 2010, 2011, 2012, 2013, 2014, 2015, 2016, 2017, 2018 |
| 28 | Estonia | 2014, 2015, 2016, 2017 |
| 29 | Fiji | 1990, 1991, 1992, 1993, 1995, 1996, 1998, 1999, 2000, 2001, 2003, 2004, 2006, 2007, 2008, 2009, 2010, 2011, 2016 |
| 30 | Finland | 2012 |
| 31 | France | 2008, 2009, 2010, 2011, 2012, 2013, 2014, 2015, 2016, 2017 |
| 32 | French Guiana | 1990, 1991, 1992, 1993, 1994, 1995, 1996, 1997, 1998, 1999, 2000, 2001, 2002, 2003, 2004, 2005, 2006, 2007, 2008, 2009, 2010, 2011, 2012, 2013, 2014, 2015, 2016, 2017, 2018 |
| 33 | Germany | 2008, 2009, 2010, 2011, 2012, 2013, 2014, 2015, 2016, 2017 |
| 34 | Greece | 2013, 2014, 2015, 2016, 2017 |
| 35 | Grenada | 1990, 1991, 1992, 1993, 1994, 1995, 1996, 1997, 1998, 1999, 2000, 2001, 2002, 2003, 2004, 2006, 2008, 2009, 2010, 2011, 2012, 2013, 2014, 2015, 2016, 2017, 2018 |
| 36 | Guadeloupe | 1990, 1991, 1992, 1995, 1996, 1999, 2000, 2002, 2003, 2005, 2006, 2007, 2008, 2009, 2010, 2011, 2012, 2013, 2014, 2015, 2016, 2017, 2018 |
| 37 | Guatemala | 1990, 1991, 1992, 1993, 1994, 1995, 1996, 1997, 1998, 1999, 2000, 2001, 2002, 2003, 2004, 2005, 2006, 2007, 2008, 2009, 2010, 2011, 2012, 2013, 2014, 2015, 2016, 2017, 2018 |
| 38 | Guyana | 1990, 1992, 1994, 1998, 2000, 2001, 2002, 2003, 2004, 2005, 2006, 2007, 2008, 2009, 2010, 2011, 2012, 2013, 2014, 2015, 2016, 2017, 2018 |
| 39 | Haiti | 2000, 2012, 2015 |
| 40 | Honduras | 1990, 1991, 1992, 1993, 1994, 1995, 1996, 1997, 1998, 1999, 2000, 2001, 2002, 2003, 2004, 2005, 2006, 2007, 2008, 2009, 2010, 2011, 2012, 2013, 2014, 2015, 2016, 2017, 2018 |
| 41 | Hungary | 2008, 2009, 2010, 2011, 2012, 2013, 2014, 2015, 2016, 2017 |
| 42 | Iceland | 2017 |
| 43 | India | 1991, 1992, 1993, 1994, 1995, 1996, 1997, 1998, 1999, 2000, 2001, 2002, 2003, 2004, 2005, 2006, 2007, 2008, 2009, 2010, 2011, 2012, 2013, 2014, 2015, 2016, 2017 |
| 44 | Indonesia | 1990, 1991, 1992, 1993, 1994, 1995, 1996, 1997, 1998, 1999, 2000, 2001, 2002, 2003, 2004, 2005, 2006, 2007, 2008, 2009, 2010, 2011, 2012, 2013, 2014, 2015, 2016, 2017 |
| 45 | Ireland | 2012, 2013, 2014, 2015, 2016, 2017 |
| 46 | Italy | 2008, 2009, 2010, 2011, 2012, 2013, 2014, 2015, 2016, 2017 |
| 47 | Jamaica | 1990, 1991, 1992, 1994, 1995, 1996, 1997, 1998, 1999, 2000, 2001, 2002, 2003, 2004, 2005, 2006, 2007, 2008, 2009, 2010, 2011, 2012, 2013, 2014, 2015, 2016, 2017, 2018 |
| 48 | Japan | 1995, 1996, 1997, 1998, 1999, 2000, 2001, 2002, 2003, 2004, 2005, 2006, 2007, 2008, 2009, 2010, 2011 |
| 49 | Kiribati | 2002, 2003, 2008, 2009, 2010, 2016 |
| 50 | Laos | 1990, 1991, 1992, 1993, 1994, 1995, 1996, 1997, 1998, 1999, 2000, 2001, 2002, 2003, 2004, 2005, 2006, 2007, 2008, 2009, 2010, 2011, 2012, 2016, 2017 |
| 51 | Latvia | 2010, 2011, 2012, 2013, 2014, 2015, 2016, 2017 |
| 52 | Lithuania | 2011, 2013, 2014, 2015, 2016, 2017 |
| 53 | Luxembourg | 2010, 2011, 2016 |
| 54 | Malaysia | 1990, 1991, 1992, 1993, 1994, 1995, 1996, 1997, 1998, 1999, 2000, 2001, 2002, 2003, 2004, 2005, 2006, 2007, 2008, 2009, 2010, 2011, 2012, 2013, 2014, 2015, 2016, 2017 |
| 55 | Maldives | 1998, 1999, 2000, 2001, 2002, 2003, 2004, 2005, 2006, 2007, 2008, 2009, 2010, 2011, 2012, 2013, 2014, 2015, 2016, 2017 |
| 56 | Malta | 2010, 2015, 2016, 2017 |
| 57 | Marshall Islands | 1991, 2011 |
| 58 | Martinique | 1990, 1992, 1995, 1996, 1997, 1998, 1999, 2000, 2001, 2002, 2003, 2005, 2006, 2007, 2008, 2009, 2010, 2011, 2012, 2013, 2014, 2015, 2016, 2017, 2018 |
| 59 | Mexico | 1990, 1991, 1992, 1993, 1994, 1995, 1996, 1997, 1998, 1999, 2000, 2001, 2002, 2003, 2004, 2005, 2006, 2007, 2008, 2009, 2010, 2011, 2012, 2013, 2014, 2015, 2016, 2017, 2018 |
| 60 | Montserrat | 1994, 1995, 1996, 2000, 2001, 2002, 2003, 2008, 2011, 2012, 2013, 2014, 2016 |
| 61 | Myanmar | 1990, 1991, 1992, 1993, 1994, 1995, 1996, 1997, 1998, 1999, 2000, 2001, 2002, 2003, 2004, 2005, 2006, 2007, 2008, 2009, 2010, 2011, 2012, 2013, 2014, 2015, 2016, 2017 |
| 62 | Nauru | 2003, 2008 |
| 63 | Nepal | 2006, 2007, 2008, 2009, 2010, 2011, 2012, 2013, 2014, 2015, 2016, 2017 |
| 64 | Netherlands Antilles | 1990, 1991, 1992, 1993, 2005, 2008, 2009, 2010, 2011, 2012, 2013, 2014, 2015 |
| 65 | New Caledonia | 1990, 1991, 1992, 1993, 1994, 1995, 1996, 1997, 1998, 1999, 2000, 2001, 2002, 2003, 2004, 2005, 2006, 2007, 2008, 2009, 2010, 2011, 2012, 2013, 2016, 2017 |
| 66 | New Zealand | 1996, 1998, 1999, 2000, 2008, 2009, 2010, 2011 |
| 67 | Nicaragua | 1990, 1991, 1992, 1993, 1994, 1995, 1996, 1997, 1998, 1999, 2000, 2001, 2002, 2003, 2004, 2005, 2006, 2007, 2008, 2009, 2010, 2011, 2012, 2013, 2014, 2015, 2016, 2017, 2018 |
| 68 | Niue | 1991, 1996, 2008, 2016 |
| 69 | Norway | 2012, 2013, 2014, 2015, 2016, 2017 |
| 70 | Palau | 1995, 1998, 1999, 2000, 2001, 2002, 2003, 2004, 2006, 2007, 2008, 2009, 2010, 2011, 2014, 2015, 2016 |
| 71 | Panama | 1993, 1994, 1995, 1996, 1997, 1998, 1999, 2000, 2001, 2002, 2003, 2004, 2005, 2006, 2007, 2008, 2009, 2010, 2011, 2012, 2013, 2014, 2015, 2016, 2017, 2018 |
| 72 | Papua New Guinea | 1991, 2002, 2009, 2016 |
| 73 | Paraguay | 1999, 2000, 2001, 2002, 2003, 2004, 2005, 2006, 2007, 2008, 2009, 2010, 2011, 2012, 2013, 2014, 2015, 2016, 2017, 2018 |
| 74 | Peru | 1990, 1991, 1992, 1993, 1994, 1995, 1996, 1997, 1998, 1999, 2000, 2001, 2002, 2003, 2004, 2005, 2006, 2007, 2008, 2009, 2010, 2011, 2012, 2013, 2014, 2015, 2016, 2017, 2018 |
| 75 | Philippines | 1990, 1991, 1992, 1993, 1994, 1995, 1996, 1997, 1998, 1999, 2000, 2001, 2002, 2003, 2004, 2005, 2006, 2007, 2008, 2009, 2010, 2011, 2012, 2013, 2014, 2015, 2016, 2017 |
| 76 | Poland | 2008, 2009, 2010, 2011, 2012, 2013, 2014, 2015, 2016, 2017 |
| 77 | Portugal | 2015, 2016, 2017 |
| 78 | Puerto Rico | 1990, 1991, 1992, 1993, 1994, 1995, 1996, 1997, 1998, 1999, 2000, 2001, 2002, 2003, 2004, 2005, 2006, 2007, 2008, 2009, 2010, 2011, 2012, 2013, 2014, 2015, 2016, 2017 |
| 79 | Romania | 2008, 2011, 2012, 2013, 2014, 2015, 2016, 2017 |
| 80 | Saint Kitts and Nevis | 1991, 1993, 1994, 1995, 1996, 1999, 2000, 2001, 2002, 2003, 2004, 2006, 2008, 2009, 2010, 2011, 2012, 2013, 2014, 2015, 2016, 2017, 2018 |
| 81 | Saint Lucia | 1990, 1991, 1993, 1995, 1996, 1997, 1998, 1999, 2001, 2002, 2003, 2004, 2005, 2006, 2007, 2008, 2009, 2010, 2011, 2012, 2013, 2014, 2015, 2016, 2017, 2018 |
| 82 | Saint Vincent and the Grenadines | 1990, 1991, 1992, 1993, 1994, 1995, 1996, 1997, 1998, 1999, 2000, 2001, 2002, 2003, 2004, 2005, 2006, 2007, 2008, 2009, 2010, 2011, 2012, 2013, 2014, 2015, 2016, 2017, 2018 |
| 83 | Samoa | 1991, 1992, 1993, 1995, 1996, 1997, 1998, 2000, 2001, 2002, 2004, 2005, 2006, 2007, 2008, 2016 |
| 84 | Singapore | 1990, 1991, 1992, 1993, 1994, 1995, 1996, 1997, 1998, 1999, 2000, 2001, 2002, 2003, 2004, 2005, 2006, 2007, 2008, 2009, 2010, 2011, 2012, 2013, 2014, 2015, 2016, 2017 |
| 85 | Slovakia | 2012, 2013, 2015, 2016, 2017 |
| 86 | Slovenia | 2008, 2009, 2010, 2011, 2012, 2013, 2014, 2015, 2016, 2017 |
| 87 | Solomon Islands | 1995, 1996, 2002, 2008, 2016 |
| 88 | South Korea | 2005, 2006, 2007, 2009, 2010, 2011, 2012, 2013, 2014, 2015, 2016 |
| 89 | Spain | 2009, 2015, 2016, 2017 |
| 90 | Sri Lanka | 1990, 1991, 1992, 1993, 1994, 1995, 1996, 1997, 1998, 1999, 2000, 2001, 2002, 2003, 2004, 2005, 2006, 2007, 2008, 2009, 2010, 2011, 2012, 2013, 2014, 2015, 2016, 2017 |
| 91 | Suriname | 1990, 1991, 1992, 1993, 1994, 1995, 1996, 1997, 1998, 1999, 2000, 2001, 2002, 2003, 2004, 2005, 2006, 2007, 2008, 2009, 2010, 2011, 2012, 2013, 2014, 2015, 2016, 2017 |
| 92 | Sweden | 2011, 2012, 2013, 2014, 2015, 2016, 2017 |
| 93 | Thailand | 1990, 1991, 1992, 1993, 1994, 1995, 1996, 1997, 1998, 1999, 2000, 2001, 2002, 2003, 2004, 2005, 2006, 2007, 2008, 2009, 2010, 2011, 2012, 2013, 2014, 2015, 2016, 2017 |
| 94 | Timor-Leste | 2004, 2005, 2006, 2007, 2008, 2009, 2010, 2011, 2012, 2013, 2014, 2015, 2016, 2017 |
| 95 | Tokelau | 1991, 1996, 2001 |
| 96 | Tonga | 1990, 1991, 1992, 1993, 1994, 1996, 1998, 2003, 2004, 2007, 2008, 2009, 2010, 2014, 2015 |
| 97 | Trinidad and Tobago | 1990, 1991, 1992, 1993, 1994, 1995, 1996, 1997, 1998, 1999, 2000, 2001, 2002, 2003, 2004, 2005, 2006, 2007, 2008, 2009, 2010, 2011, 2012, 2013, 2014, 2015, 2016, 2017, 2018 |
| 98 | Tuvalu | 1991, 1992, 1998 |
| 99 | United Kingdom | 2008, 2009, 2010, 2011, 2013, 2014, 2015, 2016, 2017 |
| 100 | United States | 1990, 1991, 1992, 1993, 1994, 1995, 2001, 2002, 2003, 2006, 2007, 2009, 2010, 2011, 2012, 2013, 2014, 2015, 2016, 2017, 2018 |
| 101 | Uruguay | 2015, 2016 |
| 102 | Vanuatu | 1990, 1991, 1992, 1993, 1994, 1995, 1996, 1998, 1999, 2003, 2004, 2005, 2006, 2007, 2008, 2009, 2010, 2011, 2016 |
| 103 | Venezuela | 1990, 1991, 1992, 1993, 1994, 1995, 1996, 1997, 1998, 1999, 2000, 2001, 2002, 2003, 2004, 2005, 2006, 2007, 2008, 2009, 2010, 2011, 2012, 2013, 2014, 2015, 2016, 2017, 2018 |
| 104 | Vietnam | 1990, 1991, 1992, 1993, 1994, 1995, 1996, 1997, 1998, 1999, 2000, 2001, 2002, 2003, 2004, 2005, 2006, 2007, 2008, 2009, 2010, 2011, 2012, 2013, 2014, 2015, 2016, 2017 |
