# Supplementary material for: Annual global dengue dynamics are related to multi-source factors revealed by a machine learning prediction analysis
Source: PLoS Negl Trop Dis. 2025 Jun 25;19(6):e0013232. doi: 10.1371/journal.pntd.0013232 (PMC12221171; doi:10.1371/journal.pntd.0013232)
Supplement: S2 Table — (DOCX) [file pntd.0013232.s002.docx]

**S2 Table. Hyperparameters of the machine learning models.**

| **Model** | **Hyperparameters** |
| --- | --- |
| Random forest | n_estimators = 200; the other hyper parameters were set to default values in RandomForestRegressor in sklearn package. |
| XGBoost | objective="reg:squarederror"; n_estimators=200; learning_rate=0.01; the other hyperparameters were set to default values in XGBRegressor in xgboost package. |
| MLP | hidden_size = 30;  num_hidden_layers= 2;  learning_rate = 0.001;  num_epochs = 200;  batch_size = 200;  Adam as the optimizer;  MSE as the loss function. |
| SVR | The hyperparameters were set to default values in SVR in sklearn package. |
