# Supplementary material for: Annual global dengue dynamics are related to multi-source factors revealed by a machine learning prediction analysis
Source: PLoS Negl Trop Dis. 2025 Jun 25;19(6):e0013232. doi: 10.1371/journal.pntd.0013232 (PMC12221171; doi:10.1371/journal.pntd.0013232)
Supplement: S3 Table — (DOCX) [file pntd.0013232.s003.docx]

**S3 Table. Performance of the models through multiple imputation method (Four folds cross validation results)**

| Models | Baseline | | | Multi-variable | | |
| --- | --- | --- | --- | --- | --- | --- |
|  | RMSE | MSE | R^2^ | RMSE | MSE | R^2^ |
| Random forest | 0.5367 | 0.2902 | 0.7058 | 0.4803 | 0.2330 | 0.7635 |
| XGBoost | 0.5361 | 0.2890 | 0.7074 | 0.4977 | 0.2490 | 0.7479 |
| MLP | 0.6547 | 0.4292 | 0.5670 | 0.5076 | 0.2586 | 0.7387 |
| SVR | 0.6466 | 0.4191 | 0.5768 | 0.5291 | 0.2809 | 0.7162 |

Note: The baseline model only used historical cases features; the multi-variable model used nine categories of features including historical cases, climate, anemia, population, air travel, vector, forest, serotype and socioeconomic factors.
