# Supplementary material for: Annual global dengue dynamics are related to multi-source factors revealed by a machine learning prediction analysis
Source: PLoS Negl Trop Dis. 2025 Jun 25;19(6):e0013232. doi: 10.1371/journal.pntd.0013232 (PMC12221171; doi:10.1371/journal.pntd.0013232)
Supplement: S4 Table — (DOCX) [file pntd.0013232.s004.docx]

**S4 Table. Performance of the models through missing indicator method (Four folds cross validation results)**

| Models | Baseline | | | Multi-variable | | |
| --- | --- | --- | --- | --- | --- | --- |
|  | RMSE | MSE | R^2^ | RMSE | MSE | R^2^ |
| Random forest | 0.5178 | 0.2720 | 0.7236 | 0.4623 | 0.2165 | 0.7801 |
| XGBoost | 0.5149 | 0.2675 | 0.7287 | 0.4844 | 0.2362 | 0.7608 |
| MLP | 0.6519 | 0.4258 | 0.5700 | 0.5458 | 0.3032 | 0.6914 |
| SVR | 0.6576 | 0.4337 | 0.5617 | 0.5344 | 0.2862 | 0.7114 |

Note: The baseline model only used historical cases features; the multi-variable model used nine categories of features including historical cases, climate, anemia, population, air travel, vector, forest, serotype and socioeconomic factors.
