# Supplementary material for: Annual global dengue dynamics are related to multi-source factors revealed by a machine learning prediction analysis
Source: PLoS Negl Trop Dis. 2025 Jun 25;19(6):e0013232. doi: 10.1371/journal.pntd.0013232 (PMC12221171; doi:10.1371/journal.pntd.0013232)
Supplement: S5 Table — (DOCX) [file pntd.0013232.s005.docx]

**S5 Table. The impact of removing various features on model performance (evaluated by mean MSE difference)**

|  | Historical case | Anemia | Air travel | Population | Vector | Climate | Serotype | Socioeconomic | Forest |
| --- | --- | --- | --- | --- | --- | --- | --- | --- | --- |
| ΔMSE | 0.0363 | 0.0195 | 0.0311 | 0.0882 | 0.0044 | 0.0784 | 0.0019 | 0.0277 | 0.0227 |
