# Supplementary material for: Annual global dengue dynamics are related to multi-source factors revealed by a machine learning prediction analysis
Source: PLoS Negl Trop Dis. 2025 Jun 25;19(6):e0013232. doi: 10.1371/journal.pntd.0013232 (PMC12221171; doi:10.1371/journal.pntd.0013232)
Supplement: S1 Fig — (PDF) [file pntd.0013232.s006.pdf]

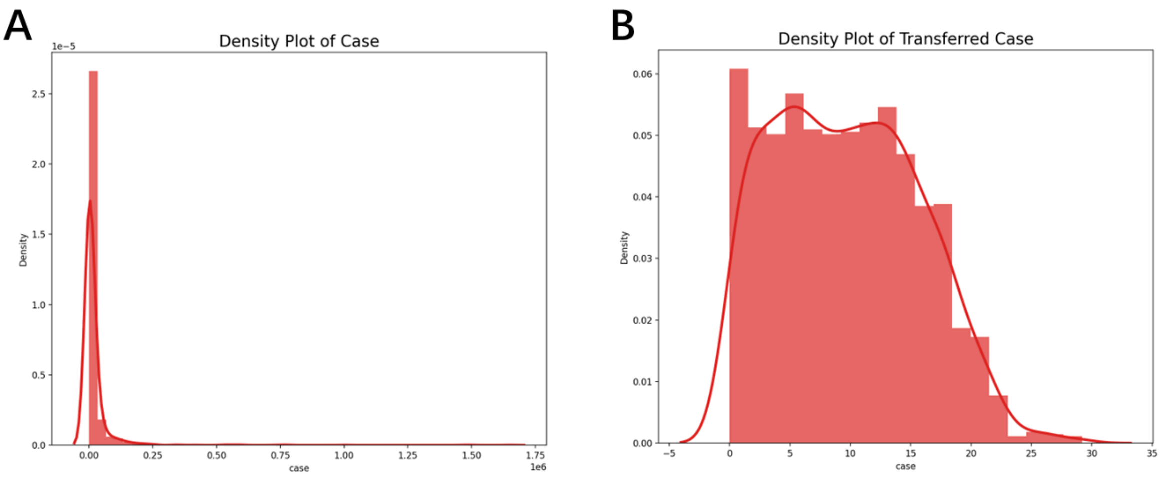

S1 Fig. Distribution of the cases data. A) displays the density of original cases data; B) displays the density of transformed cases data.
