# Supplementary material for: Annual global dengue dynamics are related to multi-source factors revealed by a machine learning prediction analysis
Source: PLoS Negl Trop Dis. 2025 Jun 25;19(6):e0013232. doi: 10.1371/journal.pntd.0013232 (PMC12221171; doi:10.1371/journal.pntd.0013232)
Supplement: S2 Fig — (PDF) [file pntd.0013232.s007.pdf]

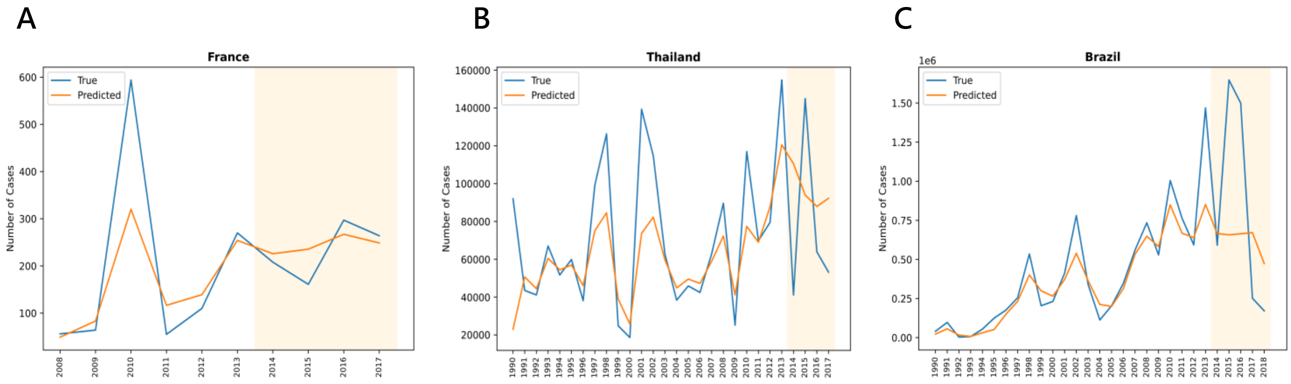

S2 Fig. The number of true cases and cases predicted by the model in three representative regions. The added orange shading represents the results of the test set. A) results of France; B) results of Thailand; C) results of Brazil.
