# Supplementary material for: Annual global dengue dynamics are related to multi-source factors revealed by a machine learning prediction analysis
Source: PLoS Negl Trop Dis. 2025 Jun 25;19(6):e0013232. doi: 10.1371/journal.pntd.0013232 (PMC12221171; doi:10.1371/journal.pntd.0013232)
Supplement: S3 Fig — (PDF) [file pntd.0013232.s008.pdf]

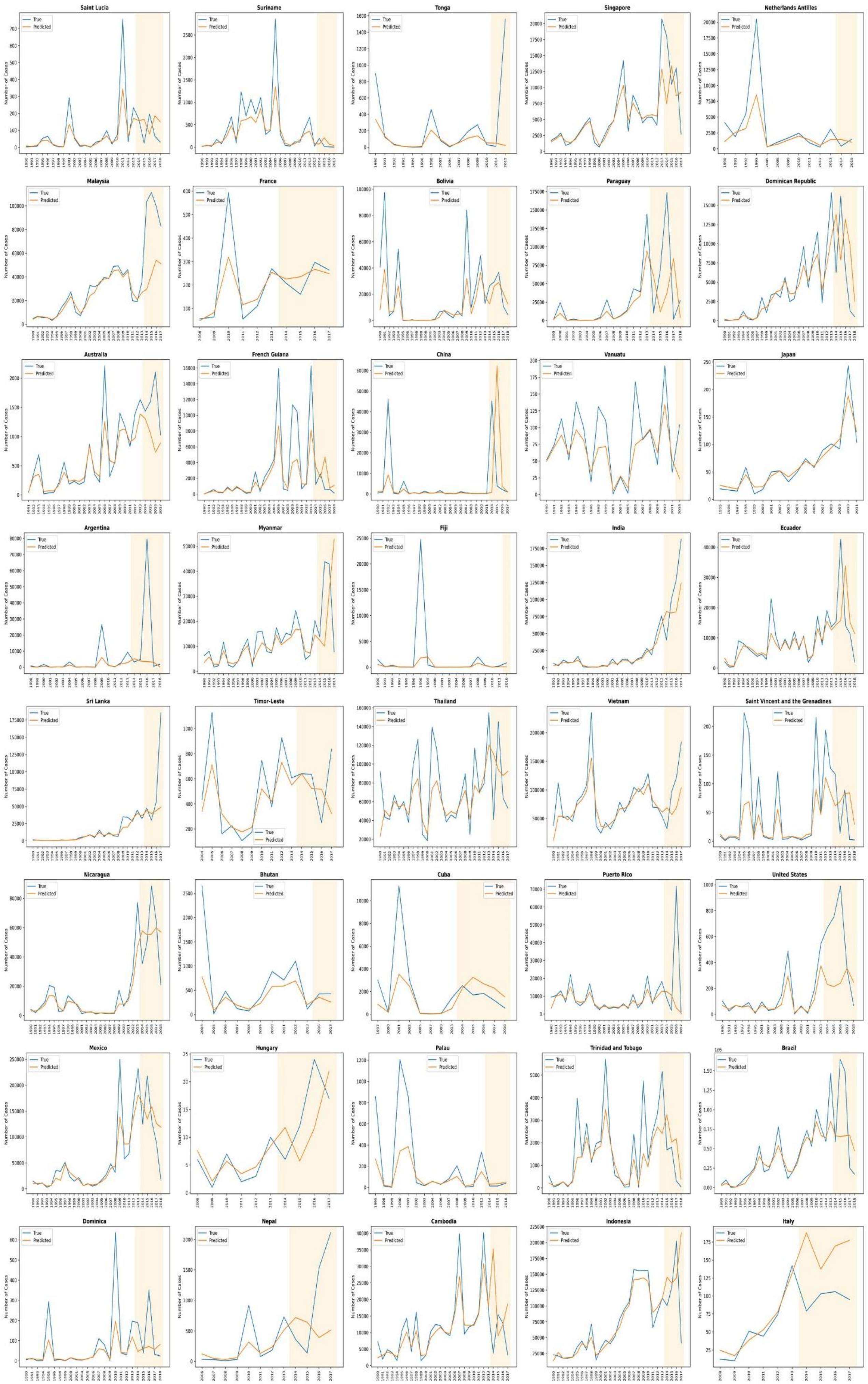
Saint Lucia
Suriname
Tonga
Singapore
Netherlands Antilles
Malaysia
France
Bolivia
Paraguay
Dominican Republic
Australia
French Guiana
China
Vanuatu
Japan
Argentina
Myanmar
Fiji
India
Ecuador
Sri Lanka
Timor-Leste
Thailand
Vietnam
Saint Vincent and the Grenadines
Nicaragua
Bhutan
Cuba
Puerto Rico
United States
Mexico
Hungary
Palau
Trinidad and Tobago
Brazil
Dominica
Nepal
Cambodia
Indonesia
Italy
True
Predicted
Number of Cases

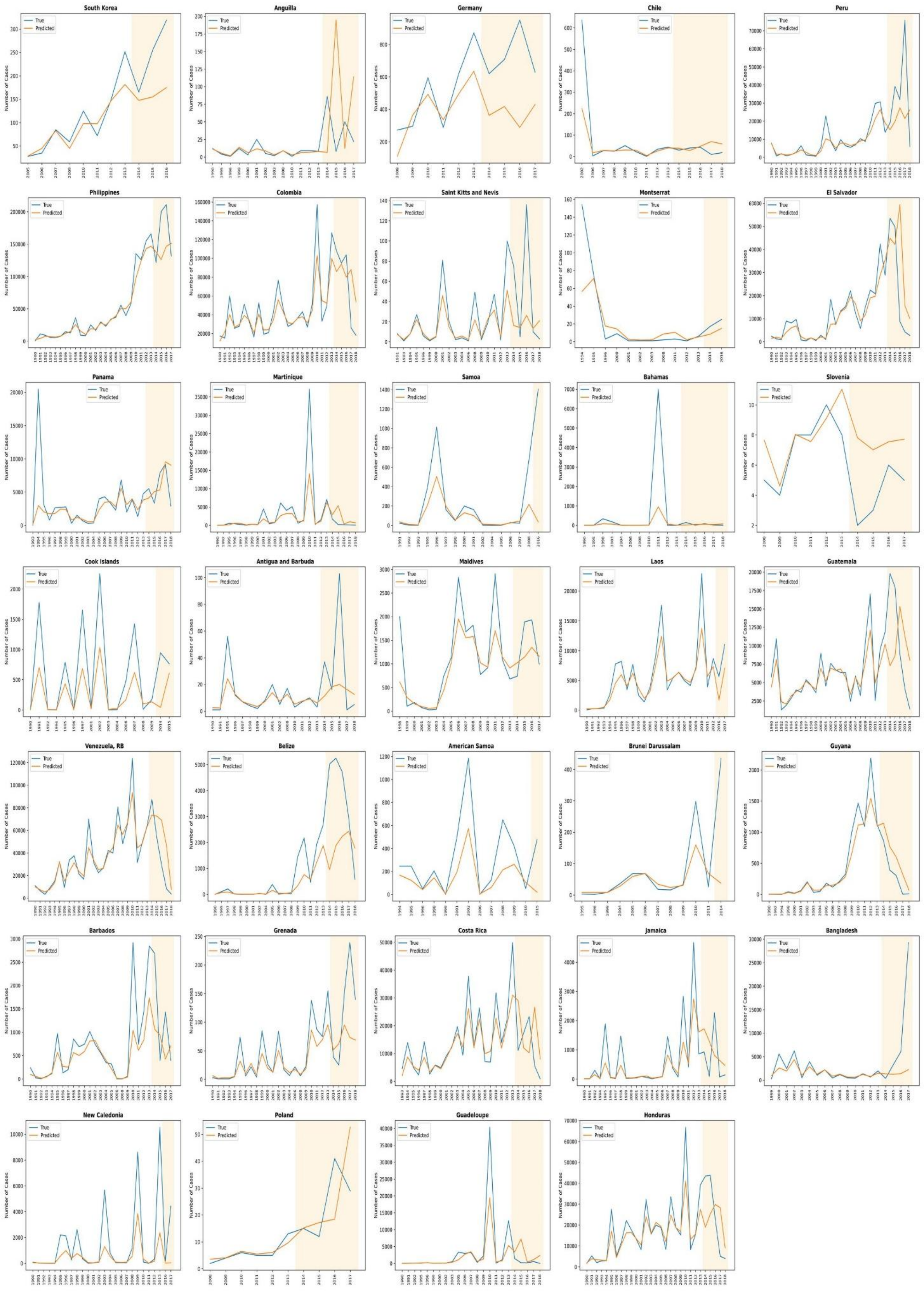
South Korea
Anguilla
Germany
Chile
Peru
Philippines
Colombia
Saint Kitts and Nevis
Montserrat
El Salvador
Panama
Martinique
Samoa
Bahamas
Slovenia
Cook Islands
Antigua and Barbuda
Maldives
Laos
Guatemala
Venezuela, RB
Belize
American Samoa
Brunei Darussalam
Guyana
Barbados
Grenada
Costa Rica
Jamaica
Bangladesh
New Caledonia
Poland
Guadeloupe
Honduras
True
Predicted
Number of Cases

**S3 Fig. The number of true cases and cases predicted by the model in regions (data available in no less than 10 years).** The added orange shading represents the results of the test set.
