# Supplementary material for: Annual global dengue dynamics are related to multi-source factors revealed by a machine learning prediction analysis
Source: PLoS Negl Trop Dis. 2025 Jun 25;19(6):e0013232. doi: 10.1371/journal.pntd.0013232 (PMC12221171; doi:10.1371/journal.pntd.0013232)
Supplement: S4 Fig — (PDF) [file pntd.0013232.s009.pdf]

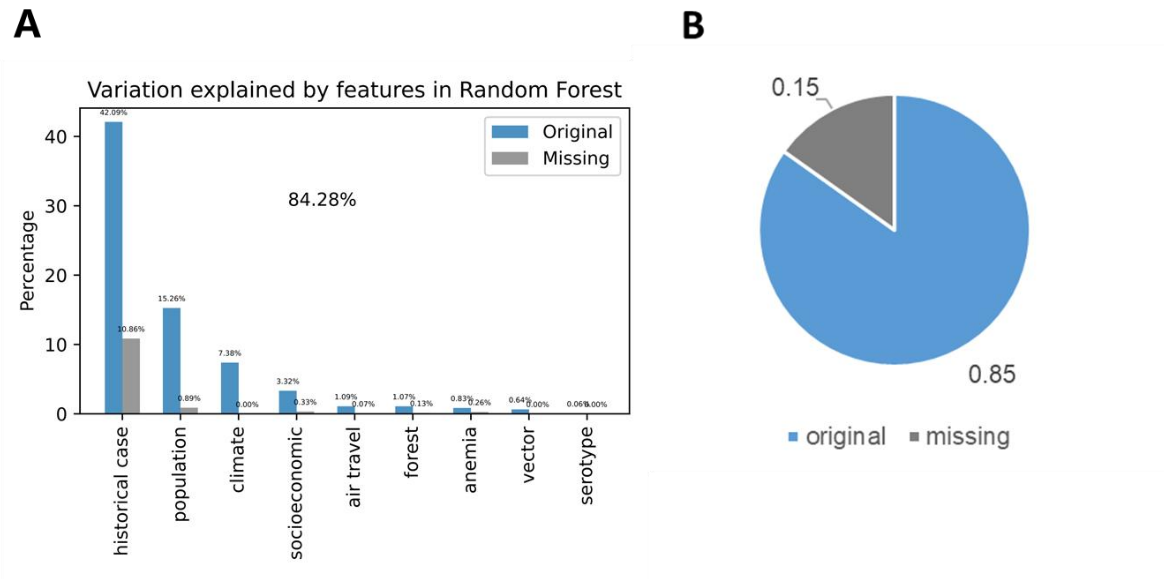

S4 Fig. Variation explained by all the categories of the features. A) displays variation explained by the nine categories of the features; B) displays the proportion of variation explained by original and missing data.
