# Supplementary material for: Annual global dengue dynamics are related to multi-source factors revealed by a machine learning prediction analysis
Source: PLoS Negl Trop Dis. 2025 Jun 25;19(6):e0013232. doi: 10.1371/journal.pntd.0013232 (PMC12221171; doi:10.1371/journal.pntd.0013232)
Supplement: S7 Fig — (PDF) [file pntd.0013232.s012.pdf]

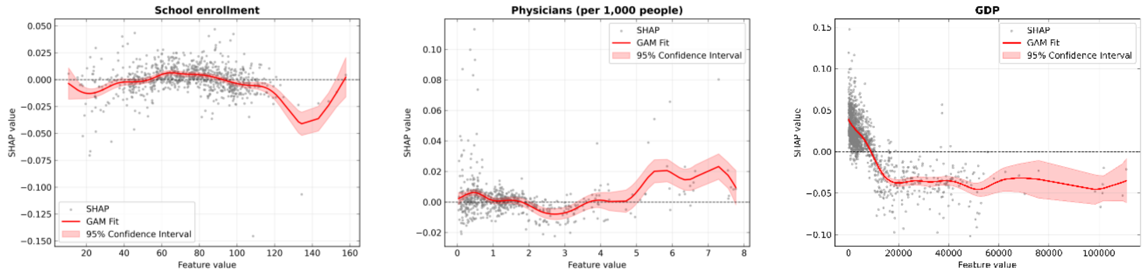

S7 Fig. Response curves of socioeconomic features. For each subplot, the horizontal axis represents the feature values, while the vertical axis represents the SHAP values. Gray dots represent the sample points, the solid red line represents the curve fitted by GAM, and the shaded area represents the 95% confidence interval.
