# Supplementary material for: Annual global dengue dynamics are related to multi-source factors revealed by a machine learning prediction analysis
Source: PLoS Negl Trop Dis. 2025 Jun 25;19(6):e0013232. doi: 10.1371/journal.pntd.0013232 (PMC12221171; doi:10.1371/journal.pntd.0013232)
Supplement: S9 Fig — (PDF) [file pntd.0013232.s014.pdf]

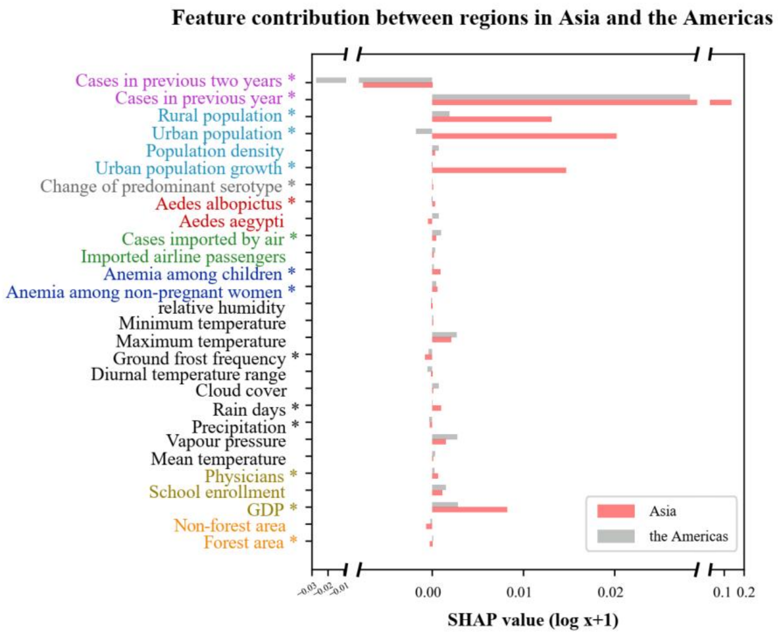

S9 Fig. Comparison of feature contribution between regions in Asia and the Americas. The multi-source different categories of features are represented with different colored fonts. Red and grey bars represent results of regions in Asia and the Americas, respectively. Asterisks indicate statistically significant results ($p<0.05$).
